# ^18^F-fluoro-deoxyglucose positron emission tomography/computed tomography scan findings in Rosai-Dorfman disease with IgG4-positive plasma cell infiltration mimicking breast malignancy: a case report and literature review

**DOI:** 10.1186/1752-1947-6-411

**Published:** 2012-11-30

**Authors:** Liping Fu, Mei Liu, Zhigang Song, Baixuan Xu, Jiahe Tian

**Affiliations:** 1 Department of Nuclear Medicine, the Chinese PLA General Hospital and PLA Medical School, Fuxing Road 28, Beijing, 100853, China; 2 Department of Pathology, the Chinese PLA General Hospital and PLA Medical School, Fuxing Road 28, Beijing, 100853, China

**Keywords:** Rosai-Dorfman disease, IgG4-related sclerosing disease, Amyloidosis, Position emission tomography

## Abstract

**Introduction:**

Rosai-Dorfman disease, also known as sinus histiocytosis with massive lymphadenopathy, is a rare benign disorder characterized histologically by lymphatic sinus dilatation due to histiocyte proliferation. Rosai-Dorfman disease accompanied by IgG4^+^ plasma cell infiltration is an even rarer situation. To the best of our knowledge, no imaging report of fluoro-deoxyglucose positron emission tomography/computed tomography findings of Rosai-Dorfman disease with IgG4^+^ plasma cell infiltration has been published, although a series of pathological research has focused on this phenomenon.

**Case presentation:**

We reviewed the ^18^F-fluoro-deoxyglucose positron emission tomography/computed tomography scan of a 78-year-old Chinese woman with a solid mass that was found in her right breast during a health checkup. ^18^F-fluoro-deoxyglucose positron emission tomography/computed tomography showed a hypermetabolic nodule in her right breast and slightly heterogeneous increased fluoro-deoxyglucose uptake of the pulmonary nodules, which were histologically proven to be mammary Rosai-Dorfman disease with IgG4^+^ plasma cell infiltration and pulmonary amyloidosis, respectively. A literature review was performed to gather information on this rare disease process.

**Conclusions:**

Although distinguishing benign lymphoplasmacytic proliferation from malignancy may be difficult with ^18^F-fluoro-deoxyglucose positron emission tomography/computed tomography in light of the pattern and intensity of fluoro-deoxyglucose uptake, our case highlights that whole-body positron emission tomography/computed tomography imaging not only can display the extent of the disease to help complete staging but also can provide functional information about disease activity to guide biopsy.

## Introduction

Rosai-Dorfman disease (RDD), also known as sinus histiocytosis with massive lymphadenopathy, is a rare benign disorder characterized histologically by lymphatic sinus dilatation due to histiocyte proliferation. The disease was first described by Destombes in 1965 [1] and was recognized as a distinct clinicopathological entity by Rosai and Dorfman in 1969 [2]. The etiology of this disease remains unclear; speculation has centered on a histiocytic reaction induced by cytokines or an as-yet-unidentified infection [3]. Clinically, RDD affects mostly children and young adults, who may present with fever, chills, and signs of systemic illness [4]. Typically, lymphadenopathy of RDD involves the cervical region. However, 43% of cases are associated with extranodal involvement [5]. RDD demonstrates a broad range of clinical presentations from no symptoms to xanthomatous skin, skin nodules, regional lymphadenopathy, and visceral mass and even death if lesions infiltrate to vital organs [6,7]. Pathologically, the characterizing feature of RDD is emperipolesis, a phenomenon that presents as variable numbers of intact lymphocytes within the cytoplasm of distinctive enlarged histiocytes. Immunohistochemical staining positive for S-100 and CD-68 protein and negative for CD1a is specific to and a prerequisite for the diagnosis of RDD [3,5,8].

IgG4-related sclerosing disease (IgG4-RSD) is a syndrome characterized by the involvement of a wide variety of tissues by lymphoplasmacytic infiltrates and sclerosis, elevated serum IgG4 titer, and increased IgG4^+^ plasma cells in tissues [9]. One or more sites, including the pancreas and retroperitoneum, could be involved in this entity. Recently, the possible relationship between RDD and IgG4-RSD was proposed [10]. However, although the diagnosis of both RDD and IgG4-RSD relies on pathological proof from the involved tissues and typical laboratory findings, integrated positron emission tomography/computed tomography (PET/CT) as a whole-body imaging tool has proven to be a valuable imaging technique for distinguishing neoplastic from benign lesions and evaluating the extent and processes of the disease. We report a case of RDD with abundant IgG4^+^ plasma cell infiltration with a fluorine-18-fluoro-deoxyglucose (^18^F-FDG) PET/CT scan. To the best of our knowledge, this is the first report of such a case.

## Case presentation

A 78-year-old Chinese woman had an isolated mass that was found in her right breast during a health checkup, and a pulmonary CT scan revealed multiple lesions in both of her lungs. She had a history of cough and expectoration for two months without fever, chest pain, dyspnea, or other complaints. During a physical examination, a nearly 2.0×2.0cm, firm nodule without tenderness was found in the lateral superior quadrant of the right breast. In a routine blood test and tumor marker screen, no remarkable abnormalities were reported. However, the anti-SS-A and anti-SS-B antibodies were positive. Our patient had high concentrations of polyclonal serum immunoglobulins (Igs): IgA 485.00mg/dL (reference range: 70 to 400mg/dL), IgG 2030.00mg/dL (reference range: 700 to 1600mg/dL), Ig light-chain kappa 432.00mg/dL (reference range: 170 to 370mg/dL), and Ig light-chain lambda 249.00mg/dL (reference range: 90 to 210mg/dL).

**Figure 1:**
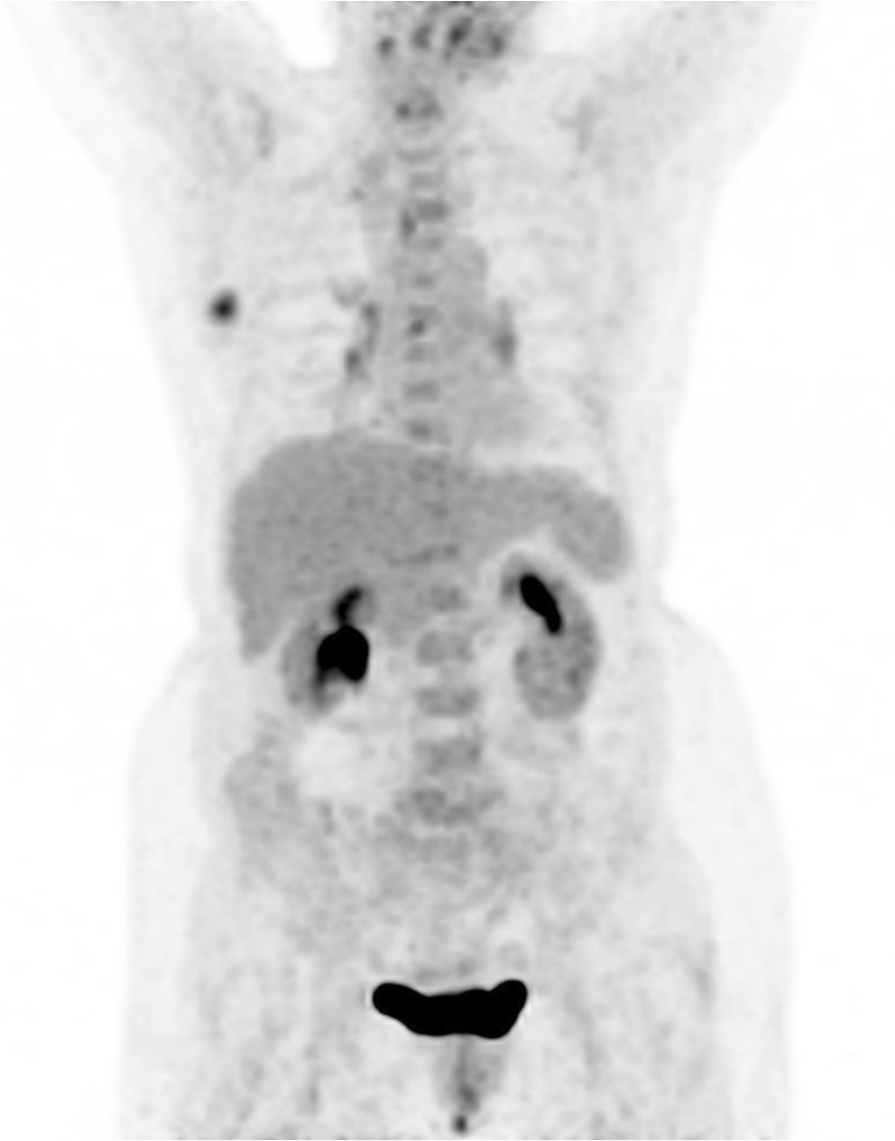
**Maximum intensity projection image of a 78-year-old woman with Rosai-Dorfman disease accompanied by IgG4^+^ plasma cell infiltration.** Besides the hypermetabolic lesion of the right breast, mild ^18^F-fluoro-deoxyglucose uptake was illustrated at the hila of the lungs and neck bilaterally.

**Figure 2:**
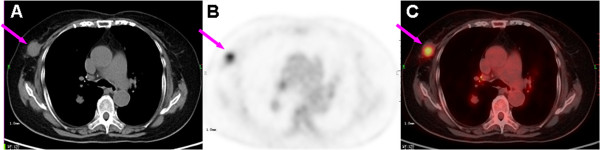
**Computed tomography (CT) (A),^18^F-fluoro-deoxyglucose positron emission tomography (FDG-PET) (B), and fused PET/CT (C) images of the mammary lesion.** Axial images show a 2.7×2.5cm, well-circumscribed, soft-tissue nodule with obvious FDG uptake (SUVmax of 7.30) in the right breast (arrows).

**Figure 3:**
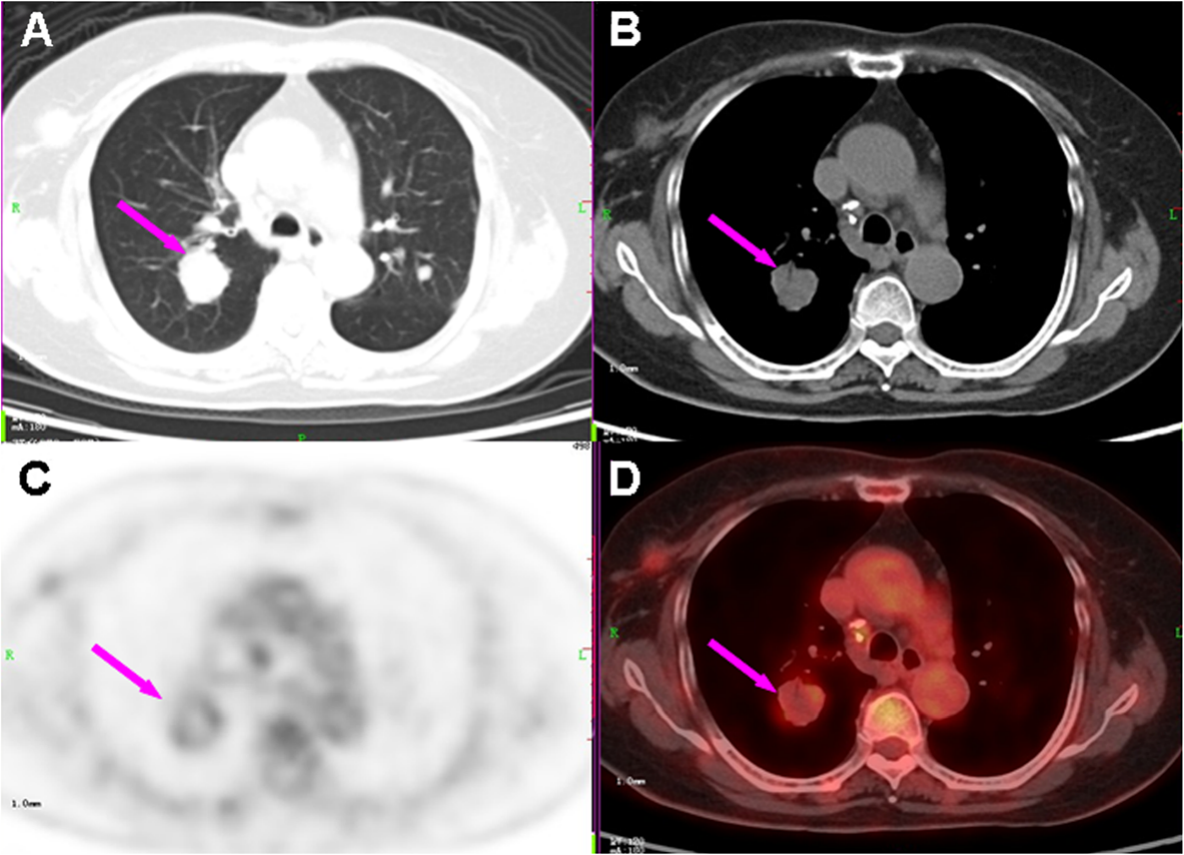
**Computed tomography (CT) lung window (A), CT mediastinal window (B),^18^F-fluoro-deoxyglucose positron emission tomography (FDG-PET) (C), and fused PET/CT (D) images of the pulmonary lesion.** Axial images show a round, relatively isodense lesion with mild ring-like FDG uptake (SUVmax of 2.96) in the right upper lobe of the lung (arrows).

Prior to a surgical resection of the mammary nodule, a whole-body PET/CT scan was suggested for the purpose of staging the possible malignant breast lesion. The scan was performed on a PET/CT system (Siemens Biograph TruePoint™ 64; Siemens Healthcare, Erlangen, Germany) at 60 minutes after intravenous injection of 6.5mCi (240MBq) of ^18^F-FDG and covered the range of the bottom of the skull to the mid thigh. On the PET maximum intensity projection image (Figure 1), an obviously hypermetabolic nodule in the right breast and slightly increased FDG uptake of the lymph nodes (LNs) at the hilus of the lungs and posterior cervical region bilaterally were revealed. On transverse images of PET/CT, the right breast lesion was a 2.3×1.9cm, round, well-circumscribed homogenous isodense nodule with a maximum standardized uptake value (SUVmax) of 7.30 (Figure 2). Multiple irregular nodules with slight to mild FDG uptake were observed in the lungs, and the largest lesion was a 2.7×2.5cm, round, well-defined solid nodule in the right lower lobe and had an SUVmax of 2.96 (Figure 3). In addition, the bilateral posterior cervical LNs were normal in size and had an SUVmax of 4.48. Given the multiple FDG-avid foci in a woman of our patient’s age, a primary breast malignant tumor lesion with pulmonary metastasis was considered first and surgery on the mammary lesion was performed three days later.

**Figure 4:**
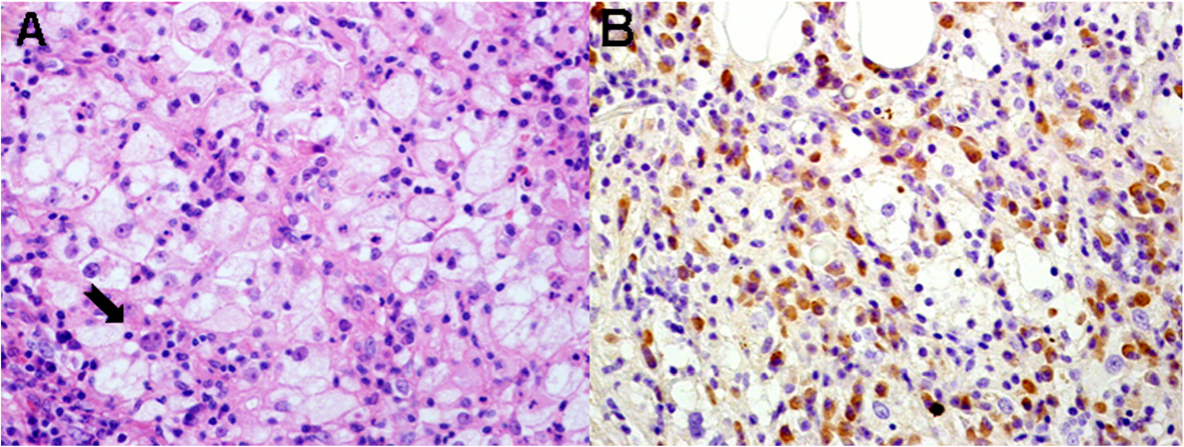
**Histopathological images and immunohistochemical staining of the mammary lesion.** (**A**) High-power photomicrograph shows distinctive histiocytes of Rosai-Dorfman disease that are large with abundant and finely granular pink cytoplasm and relatively large nuclei with open chromatin and distinct nucleoli. Engulfment of intact lymphocytes and plasma cells by the large histiocytes, a phenomenon known as emperipolesis (arrow), is shown (stain: hematoxylin-eosin; original magnification: ×400). (**B**) Immunohistochemical staining for IgG4 labels most of the plasma cells (IgG immunohistochemical stain counterstained by hematoxylin-eosin; original magnification: ×400).

Tissues of the mammary lesion were reviewed for histopathological features, and formalin-fixed and paraffin-embedded tissues were immunostained with antibodies to IgG4 (1:300, clone HP6025; Zymed, now part of Invitrogen Corporation, Carlsbad, CA, USA), IgG (1:200, clone A57H; Dako, Glostrup, Denmark), S-100 protein (1:1000, polyclonal; Dako), and CD68 protein (1:100, clone KP1; Dako). Congo red stain was used to test amyloid deposits. Pathologically, the mammary lesion showed abundant plasma cells and sinus histiocytes, including large pale pathognomonic histiocytes that exhibited emperipolesis (Figure 4A). In addition, lymphoid follicle formation, patchy fibrosis, and obliterative phlebitis, accompanied by atrophy and loss of mammary lobules, were observed. Further immunohistochemical staining showed large histiocytes positive for S-100 protein (Figure 4B) and CD-68 protein but negative for CD1a. The average number of IgG4^+^ cells was 118 per high-powered field, and the ratio of IgG4^+^ to IgG^+^ cells was 65%.

The lesion in the upper lobe of the right lung and the LN at the right carotid sheath were biopsied. Histology revealed deposition of strongly Congo red-positive materials with few histiocytes and fibrotic cell infiltration in the lung tissue. Reactive lymphoproliferation and deposition of Congo red-positive materials within the adjacent connective tissue were observed in the cervical LN.

## Discussion

The use of ^18^F-FDG PET has evolved in the last few decades and is now considered one of the mainstays of diagnosis and staging as well as of monitoring the effects of treatment in the field of oncology. However, the uptake of FDG is not specific [11]: uptake is seen not only in normal organs but also in other non-neoplastic etiologies such as inflammatory, reactive, or infectious causes. RDD, as a benign lymphoproliferative disorder, is generally believed to be FDG-avid. Clinically, RDD is commonly associated with lymphadenopathy of the cervical region, but involvement of extranodal organs such as the skin and paranasal sinuses is not rare [5]. Consequently, the isolated or widespread FDG-avid lesions of RDD can mimic malignancies and thus lead to a false image interpretation. Therefore, RDD that exhibits massive lymphadenopathy or extranodal manifestations or both should be considered in the differential diagnosis of a malignant process, such as lymphoma or metastatic tumors.

An elevated serum titer of IgG4 is a surrogate marker for the recently characterized IgG4-RSD in multiple extra-pancreatic sites with or without an accompanying pancreatic lesion [12]. Pathologically, IgG4-RSD is an inflammatory or fibrosing disease process (or both) characterized by the presence of a prominent number of IgG4^+^ plasma cells within the affected tissues [12]. Although there is no consensus about the definite cutoff limit for the number of IgG4^+^ plasma cells and the ratio of IgG4^+^ to IgG^+^ cells as the diagnostic criteria for IgG4-RSD, immunostaining shows an absolute number of IgG4^+^ cells of greater than 50 per high-powered field, and an IgG4^+^/IgG^+^ cell ratio of greater than 40% is well accepted by investigators [9].

Generally, both RDD and IgG4-RSD have been proven to be FDG-avid [13,14]. Although the mechanism of RDD and IgG4-RSD with increased FDG uptake is not certain, it has been well documented that variable expression of the glucose transporters may depend on expression of varying stress-related proteins or signaling molecules induced by activated macrophages (histiocytes) and lymphocytes [15]. In previous ^18^F-FDG PET reports, RDD revealed as hypermetabolic lesions was found within the thyroid, skin, and bones, where FDG-PET was used for staging the supposed malignancy and surveying the extent of RDD [4,16]. For IgG4-RSD, FDG-PET was suggested as an effective modality to provide extra-pancreatic biopsy sites, monitor therapy, and demonstrate relapse [14]. To the best of our knowledge, no report of FDG PET/CT findings of RDD with IgG4^+^ plasma cell infiltration has been published, although a series of pathological research has focused on this phenomenon. In our case, the hypermetabolic soft-tissue nodule in the right breast accompanied by multiple active pulmonary nodules mimicked breast malignancy with lung metastasis. Even in a review of the PET/CT images, it seemed unfeasible to discriminate the nature of FDG-avid breast RDD from carcinoma simply by the distribution pattern or intensity of the FDG accumulation. In addition, the pancreas, hepatobiliary system, and glandular organs, which are the most common sites involved in IgG4-RSD, showed no abnormal FDG accumulation in our patient. In this case, the value of ^18^F-FDG PET/CT is mainly in localizing the proper target sites for tissue biopsy and thereby confirming the diagnosis and helping to adjust the clinical treatment strategy.

It is interesting to find amyloid deposition rather than abundant histiocytes or IgG4^+^ plasma cell accumulation within pulmonary nodules. In addition, amyloidosis was still observed in the connective tissues surrounding the cervical LNs. For PET/CT images, the slight FDG uptake at the edge of the solid pulmonary nodule was different from the typical radioactivity distribution pattern of hematogenous metastatic lesions, which corresponded well to the pathological findings of diffuse amyloid deposition accompanied by few histiocytic infiltrations at the boundary of the samples. Pulmonary amyloidosis was previously reported as multiple nodules with slight FDG uptake and which were raised suspicion of metastasis from a hidden malignancy [17]. Therefore, it is suggested that, in the event of multiple irregular variable-sized nodules with the special FDG distribution pattern in both lungs, certain kinds of non-tumor diseases should be taken into diagnostic consideration. Though rare, amyloidosis ought to be kept in mind as a differential diagnosis of metastatic lesions. In fact, amyloidosis is caused by the extracellular deposition of pathological, insoluble, fibrillar proteins in organs and tissues. Secondary amyloidosis is caused by the deposition of amyloid originating from serum amyloid A, which is an acute-phase protein produced in response to inflammation and occurs most commonly among patients with chronic inflammatory diseases such as rheumatoid arthritis, juvenile rheumatoid arthritis, and inflammatory bowel disease [18]. The reason for the amyloid deposition in this case is not clear, but a plausible postulation is that it was associated with the chronic inflammatory stimulation of the mammary lesion and/or autoimmune abnormalities represented by raised serum polyclonal immunoglobulins and low titers of autoantibodies. Further studies may be needed to explore the underpinning relationship between serum amyloid A and autoimmune disease, RDD and IgG4-RSD.

## Conclusions

In summary, we report ^18^F-FDG PET/CT findings in a case of breast RDD accompanied by IgG4^+^ plasma cell infiltration. Although distinguishing benign lymphoplasmacytic proliferation from other malignant lesions may be difficult with ^18^F-FDG PET/CT in light of the pattern and intensity of FDG uptake, our case highlights that whole-body PET/CT imaging can display the extent of the disease, help to complete staging, and provide functional information about disease activity to guide biopsy and can be used to monitor the therapeutic response as well.

## Abbreviations

F-FDG: Fluorine-18-fluoro-deoxyglucose; FDG: Fluoro-deoxyglucose; Ig: Immunoglobulin; IgG4-RSD: IgG4-related sclerosing disease; LN: Lymph node; PET/CT: Positron emission tomography/computed tomography; RDD: Rosai-Dorfman disease; SUVmax: Maximum standardized uptake value.

## Competing interests

The authors declare that they have no competing interests.

## Authors’ contributions

FL was involved in collecting the data, writing the manuscript, organizing the text and PET/CT images, and conducting the literature review. LM and SZ provided the pathological photos and detailed histological information. XB obtained informed consent. TJ served as advisor to FL and was responsible for the critical revision of the manuscript. All authors read and approved the final manuscript.

## Consent

Written informed consent was obtained from the patient for publication of this case report and accompanying images. A copy of the written consent is available for review by the Editor-in-Chief of this journal.

## References

[B1] Destombes P Adenitis with lipid excess, in children or young adults, seen in the Antilles and in Mali (4 cases) Bull Soc Pathol Exot Filiales 1965 58 1169 1175 5899730

[B2] Rosai J Dorfman RF Sinus histiocytosis with massive lymphadenopathy: a newly recognized benign clinicopathological entity Arch Pathol 1969 87 63 70 5782438

[B3] Chan JK Tsang WY Uncommon syndromes of reactive lymphadenopathy Semin Oncol 1993 20 648 657 8296201

[B4] Lim R Wittram C Ferry JA Shepard JA FDG PET of Rosai-Dorfman disease of the thymus AJR 2004 182 514 14736692 10.2214/ajr.182.2.1820514

[B5] Foucar E Rosai J Dorfman R Sinus histiocytosis with massive lymphadenopathy (Rosai-Dorfman disease): review of the entity Semin Diagn Pathol 1990 7 19 73 2180012

[B6] Krishnan A Nassar A Nieh PT Rosai-Dorfman disease presenting as extra nodal renal mass Urology 2005 66 1319 16360469 10.1016/j.urology.2005.06.103

[B7] Foucar E Rosai J Dorfman RF Sinus histiocytosis with massive lymphadenopathy. An analysis of 14 deaths occurring in a patient registry Cancer 1984 54 1834 1840 10.1002/1097-0142(19841101)54:9<1834::AID-CNCR2820540911>3.0.CO;2-F 6478418

[B8] Kademani D Patel SG Prasad ML Huvos AG Shah JP Intraoral presentation of Rosai-Dorfman disease: a case report and review of the literature Oral Surg Oral Med Oral Pathol Oral Radiol Endod 2002 93 699 704 10.1067/moe.2002.123495 12142877

[B9] Divatia M Kim SA Ro JY IgG4-related sclerosing disease, an emerging entity: a review of a multi-system disease Yonsei Med J 2012 53 15 34 10.3349/ymj.2012.53.1.15 22187229 PMC3250325

[B10] Kuo TT Chen TC Lee LY Lu PH IgG4-positive plasma cells in cutaneous Rosai-Dorfman disease: an additional immunohistochemical feature and possible relationship to IgG4-related sclerosing disease J Cutan Pathol 2009 36 1069 1073 10.1111/j.1600-0560.2008.01222.x 19187110

[B11] Metser U Even-Sapir E Increased (18)F-fluorodeoxyglucose uptake in benign, nonphysiologic lesions found on whole-body positron emission tomography/computed tomography (PET/CT): accumulated data from four years of experience with PET/CT Semin Nucl Med 2007 37 206 222 10.1053/j.semnuclmed.2007.01.001 17418153

[B12] Bateman AC Deheragoda MG IgG4-related systemic sclerosing disease - an emerging and under diagnosed condition Histopathology 2009 55 373 383 10.1111/j.1365-2559.2008.03217.x 19817887

[B13] Yu JQ Zhuang H Xiu Y Talati E Alavi A Demonstration of increased FDG activity in Rosai- Dorfman disease on positron emission tomography Clin Nucl Med 2004 29 209 210 10.1097/01.rlu.0000114018.07676.15 15162996

[B14] Nguyen VX De Petris G Nguyen BD Usefulness of PET/CT imaging in systemic IgG4-related sclerosing disease. A report of three cases JOP 2011 12 297 305 21546713

[B15] Fu Y Maianu L Melbert BR Garvey WT Facilitative glucose transporter gene expression in human lymphocytes, monocytes, and macrophages: a role for GLUT isoforms 1, 3, and 5 in the immune response and foamcell formation Blood Cells Mol Dis 2004 32 182 190 10.1016/j.bcmd.2003.09.002 14757434

[B16] Tsang JS Anthony MP Wong MP Wong CS The use of FDG-PET/CT in extra nodal Rosai-Dorfman disease of bone Skeletal Radiol 2012 41 715 717 10.1007/s00256-012-1382-9 22450661

[B17] Seo JH Lee SW Ahn BC Lee J Pulmonary amyloidosis mimicking multiple metastatic lesions on F-18 FDG PET/CT Lung Cancer 2010 67 376 379 10.1016/j.lungcan.2009.11.014 20022134

[B18] Lachmann HJ Goodman HJ Gilbertson JA Gallimore JR Sabin CA Gillmore JD Hawkins PN Natural history and outcome in systemic AA amyloidosis N Engl J Med 2007 356 2361 2371 10.1056/NEJMoa070265 17554117

